# Intergenic disease-associated regions are abundant in novel transcripts

**DOI:** 10.1186/s13059-017-1363-3

**Published:** 2017-12-28

**Authors:** N. Bartonicek, M. B. Clark, X. C. Quek, J. R. Torpy, A. L. Pritchard, J. L. V. Maag, B. S. Gloss, J. Crawford, R. J. Taft, N. K. Hayward, G. W. Montgomery, J. S. Mattick, T. R. Mercer, M. E. Dinger

**Affiliations:** 1 Garvan Institute of Medical Research, Sydney, NSW Australia; 2 Department of Psychiatry, University of Oxford, Warneford Hospital, Oxford, UK; 3 Faculty of Medicine, St Vincent’s Clinical School, University of New South Wales, Sydney, NSW Australia; 4 QIMR Berghofer Medical Research Institute, Brisbane, QLD Australia; 5 Institute for Molecular Bioscience, The University of Queensland, Brisbane, QLD Australia; 6 Illumina, Inc., San Diego, CA USA; 7 Altius Institute for Biomedical Sciences, Seattle, USA

## Abstract

**Background:**

Genotyping of large populations through genome-wide association studies (GWAS) has successfully identified many genomic variants associated with traits or disease risk. Unexpectedly, a large proportion of GWAS single nucleotide polymorphisms (SNPs) and associated haplotype blocks are in intronic and intergenic regions, hindering their functional evaluation. While some of these risk-susceptibility regions encompass cis-regulatory sites, their transcriptional potential has never been systematically explored.

**Results:**

To detect rare tissue-specific expression, we employed the transcript-enrichment method CaptureSeq on 21 human tissues to identify 1775 multi-exonic transcripts from 561 intronic and intergenic haploblocks associated with 392 traits and diseases, covering 73.9 Mb (2.2%) of the human genome. We show that a large proportion (85%) of disease-associated haploblocks express novel multi-exonic non-coding transcripts that are tissue-specific and enriched for GWAS SNPs as well as epigenetic markers of active transcription and enhancer activity. Similarly, we captured transcriptomes from 13 melanomas, targeting nine melanoma-associated haploblocks, and characterized 31 novel melanoma-specific transcripts that include fusion proteins, novel exons and non-coding RNAs, one-third of which showed allelically imbalanced expression.

**Conclusions:**

This resource of previously unreported transcripts in disease-associated regions (http://gwas-captureseq.dingerlab.org) should provide an important starting point for the translational community in search of novel biomarkers, disease mechanisms, and drug targets.

**Electronic supplementary material:**

The online version of this article (doi:10.1186/s13059-017-1363-3) contains supplementary material, which is available to authorized users.

## Background

The success of genome-wide association studies (GWAS) in discovering risk loci for various traits and diseases [1–8] is yet to be matched by the identification of biological roles for these variants. The GWAS methodology inherently presents challenges to the functional annotation of individual genetic variants. The reported GWAS single nucleotide polymorphism (SNP) is rarely the causal variant for the associated trait or disease and is instead a marker for a co-inherited genomic region: the linkage disequilibrium (LD) or haplotype block (haploblock) [1, 3, 9–11]. Pinpointing the casual variant is often restricted by the limited SNP composition of the genotyping arrays, the small size of studied populations, as well as their unique haploblock makeup [12–15]. However, the technical limitations are not the main reason for a small number of GWAS-identified genes involved in formation of complex phenotypes [16–21]. The key issue is that the majority of haploblocks with GWAS SNPs do not overlap portions of the genome of known function and remain classified as intronic or intergenic [22–26].

The common presence of disease-associated loci in intronic and intergenic regions is usually attributed to potential regulatory functions of DNA sequence. Variations at a single nucleotide may influence large conformational changes of DNA structure by affecting the state of the chromatin and interactions between distant loci [25, 27–31]. Furthermore, variants at individual nucleotides can also disrupt protein–DNA or RNA–DNA interactions [32–34], altering the binding of promoters and enhancers by regulatory proteins or RNA molecules, or regulating deposition of epigenetic marks [35–42]. However, the scarce overlap of disease-associated variation with known genes is undoubtedly influenced by the incomplete annotation of the human transcriptome.

Sequencing technologies such as RNA sequencing (RNA-seq) have revolutionized our understanding of the transcriptional landscape of the human genome, though the exhaustive annotation of genes or transcripts is far from complete. In the last five years, over 10,000 novel transcribed loci have been added to the GENCODE catalogue [35, 43, 44] and the exploration of additional layers of transcriptome complexity, such as splice variants and gene fusions, is in its infancy [45–49]. Despite the initial success of RNA-seq, its well-described limitations call for novel techniques that provide higher resolution, especially in the characterization and discovery of transcripts that may be cell-specific and therefore appear to be lowly expressed in complex tissues [50–54]. This is particularly true for long non-coding RNAs (lncRNAs), which are typically expressed at orders of magnitude lower abundance than messenger RNAs (mRNAs) and require larger sequencing coverage for assembly and quantification [55–58]. This bias further impairs the detection of lncRNAs present only in specific cells, tissues or during a limited timeframe [59, 60]. To overcome this challenge, several experimental and computational methodologies have been developed [61, 62], with CaptureSeq as the most recent addition [63–65].

CaptureSeq is a method for targeted RNA-seq of transcripts expressed from specific genomic regions of interest (ROIs) [63]. The underlying principle, shared with other target-enrichment methods [66–69], is based on the hybridization of nucleic acid libraries with custom oligonucleotides, allowing for enrichment of specific RNA sequences and the consequent deeper sequencing of targeted regions [64]. This technique can detect lowly expressed transcripts with > 100 times higher sensitivity than standard RNA-seq and has previously provided the first high-resolution map of human splicing branchpoints [46, 63]. The specificity and high resolution of this method make it an ideal technique to detect transcriptional events in the proximity of intergenic GWAS SNPs.

To investigate the hypothesis that many trait- and disease-associated SNPs lie within proximity of previously unannotated transcripts, we employed CaptureSeq on transcriptomes from 21 tissues and 13 melanoma samples, targeting 561 intronic and intergenic haplotype blocks with GWAS SNPs and nine additional melanoma risk loci. Here, we report and extensively characterize 1775 transcribed loci with multi-exonic transcripts that are mostly tissue-specific and originate from the vast majority of haploblocks with GWAS SNPs, as well as 31 novel melanoma transcripts, providing an important resource to the translational community in search of targeted therapies, biomarkers, and disease mechanisms.

## Results

### Majority of intronic and intergenic haploblocks with GWAS SNPs are transcriptionally active

**Fig. 1:**
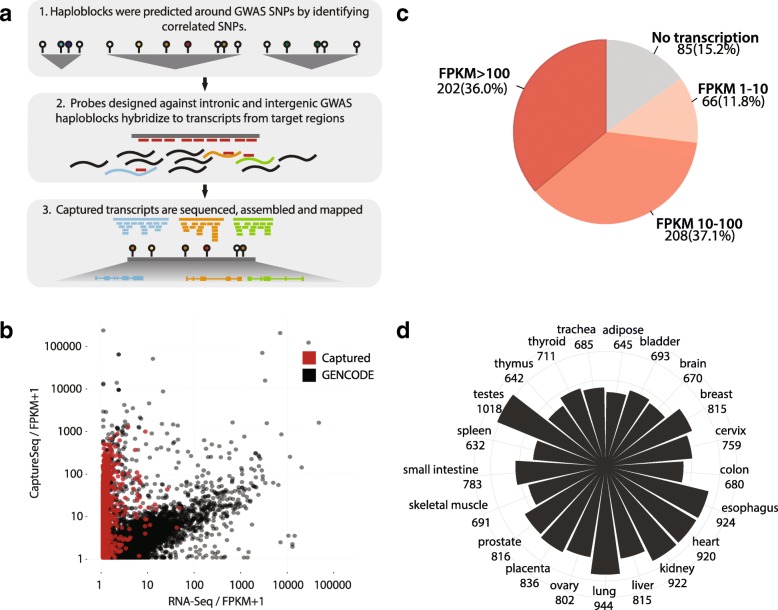
Capturing novel transcripts from intronic and intergenic GWAS regions. **a** Schematic of the experimental design. LD blocks were predicted around GWAS SNPs (*colored pins*) by identifying proxy (i.e. co-inherited) SNPs (r^2^ > 0.5) from Hapmap23 and 1000 genomes (*white pins*). Oligonucleotide probes were designed for 561 intronic and intergenic GWAS regions and hybridized to transcriptomes of 21 target tissues. The captured transcripts were sequenced, assembled, and mapped back to the genome. **b** Enrichment of captured transcripts. Expression of all captured (*red*) and non-captured (*black*) transcripts annotated in GENCODE (v.19) was compared between testis CaptureSeq sample (*y-axis*) vs testis RNA-seq from Illumina Body Atlas (*x-axis*). Correlation coefficients are 0.29 for captured transcripts and 0.55 for GENCODE genes. FPKM: fragments per kilobase of transcripts per million mapped reads. **c** Occupancy of 561 intergenic haploblocks by multi-exonic captured transcripts. The majority of haploblocks (84.8%) contain at least one transcript with FPKM > 1. **d** Counts of captured multi-exonic transcripts with FPKM > 1 across tissues

To capture previously undetected tissue-specific or lowly expressed transcripts in proximity of GWAS SNPs, we employed CaptureSeq on the transcriptomes of 21 tissues, enriching for transcription events from 561 intronic and intergenic regions, covering 73.9 Mb (2.2%) of the human genome, associated with 392 traits and diseases (Additional file 1: Table S1a). Oligonucleotide probes were designed to tile haplotype blocks with significant GWAS SNPs (339 pilot haploblocks with *p* value < 10^−5^ and 296 with *p* value < 5 × 10^–8^), while eliminating coding exons from GENCODE (v.12) or RefSeq (Fig. 1a, Additional file 1: Table S1b–e). These probes were then used as described in the CaptureSeq protocol [64] to enrich RNA from individual tissues for novel transcripts. We subsequently sequenced the transcript libraries (paired-end, 100 nt reads) and developed an analysis workflow for their de novo assembly, genome mapping, and quantification, focusing on the removal of assembly noise and lowly expressed isoforms to infer robust transcription (see “Methods”).

In order to assess the amount of potential transcriptional noise, we introduced multiple control regions to the capture design: a known gene desert on chromosome 7 and numerous intronic and exonic loci (Additional file 1: Table S1f, g). The control gene desert and introns had significantly lower odds of containing multi-exonic transcripts, 0.75 times (*p* value < 4.9 × 10^–324^, Χ^2^ test) and 0.92 times (*p* value 1.4 × 10^–14^, Χ^2^ test), respectively, which covered 10% and 12% of the control regions (Additional file 2: Figures S1a, b). The odds were increased for GENCODE exons (11.1 times, *p* value < 4.9 × 10^–324^, Χ^2^ test). On the other hand, odds of identifying single-exonic transcripts, more likely to represent spurious transcripts and assembled introns, were 4.95 times higher in gene deserts and 5.41 times in introns. In addition, the transcripts were expressed across the haploblocks in a non-random manner (Additional file 2: Figure S1c).

To avoid the larger false-positive rate for single-exonic transcripts, we focused only on transcribed loci that produced spliced transcripts (referred hereafter as “captured transcripts”). This allowed us to identify 1775 multi-exonic transcribed loci with FPKM > 1 in at least one tissue (Additional file 1: Table S2). For simplicity, these captured transcripts were separated into low, medium, and high categories based on their expression level (Fig. 1c) and assembly quality (Additional file 2: Figure S2a). Comparison to standard RNA-seq confirmed ~ 100 times enrichment of transcripts from target regions and 2.6-fold depletion of GENCODE genes (Fig. 1b). In support of the authenticity of the novel splice junctions, the majority were canonical, did not overlap repeat regions, and were not a result of multi-mapping reads (Additional file 1: Figures S2b–d). Out of 561 haploblocks, 84.8% contained at least one multi-exonic transcript, with about one-third of the transcripts expressed in each individual tissue (Fig. 1c, d). CaptureSeq methodology allowed us to identify widespread independent transcriptional activity throughout disease-associated intronic and intergenic regions.

### Genomic loci of novel transcripts bear hallmarks of active transcription

**Fig. 2:**
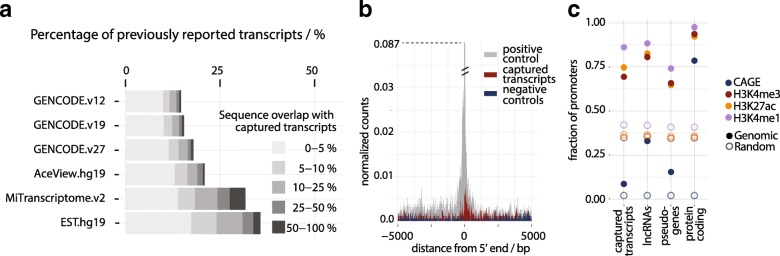
Defining properties of novel transcripts. **a** Previous observation of portions of captured transcripts in public databases. Percent of captured transcripts overlapping previously annotated transcripts in GENCODE at the time of the experiment design (v.12), GENCODE v.19 and v.27, AceView, MiTranscriptome, and the EST database. *Gray shades* indicate length overlap between the novel transcript and the previously observed sequences. **b** Aggregated data for cap analysis gene expression (CAGE) clusters, centered on the 5’ end of captured transcripts. Counts are normalized by the number of transcripts. Positive control was defined as lncRNAs transcripts with the same median of expression distribution across tissues as captured transcripts, from Illumina Body Map data. *X-axes* indicate distance from the 5’ start of transcripts in base pairs. *Y-axes* represent counts of CAGE clusters, normalized by the number of transcripts (see “Methods”). **c** Fraction of promoters of captured transcripts, lncRNAs, pseudogenes, and protein-coding genes occupied by CAGE and epigenetic marks: CAGE (*blue*), H3K4me3 (*red*), H3K27ac (*yellow*), H3K4me1 (*purple*). *Hollow circles* represent randomized controls, whereby CAGE and epigenetic peaks were randomly distributed across the genome

Having identified a multitude of captured transcripts in the haploblocks with GWAS SNPs, we investigated their sequence and genomic properties to provide further evidence for their active transcription. We first overlaid the genomic loci of the captured transcripts with known gene annotations. GENCODE v.19 as the more conservative database shared 15% of the transcripts, with the sequence overlap confined to a small portion of the capture transcript and with a growing proportion over later GENCODE versions, while the more permissive databases such as AceView and ESTs reported 20–30%, with the highest sequence overlap from MiTranscriptome [44, 48, 70, 71] (Fig. 2a). Next, we measured the coding potential of the captured transcripts with the Coding-Potential Assessment Tool (CPAT) and Coding Potential Calculator (CPC) [72, 73]. The majority of transcripts had even lower coding potential than known lncRNAs (*p* value < 2.6 × 10^–16^ CPC, *p* < 0.0001 (Kruskal–Wallis with Dunn’s multiple comparisons test) CPAT, Additional file 2: Figure S3a, b). As expected, other properties such as conservation, number of exons and isoforms were on average more similar to lncRNAs than protein-coding genes (Additional file 2: Figure S3c–e).

To evaluate whether the captured transcripts bear the typical hallmarks of expression, we matched their tissue-specific expression with cap analysis gene expression (CAGE) data from the FANTOM project and histone methylation marks from Roadmap Epigenomics [74, 75]. CAGE tags define the 5’ end of a transcribed RNA, while the investigated histone methylation marks are enriched at the sites of active transcription (H3K4me3, H3K4me1, and H3K27ac) [76–78]. Even though the transcript promoters overlapped CAGE clusters in only 8% of cases (24% over their whole region) the start sites of captured transcripts were enriched for CAGE tags compared to the genomic background (Fig. 2b, Additional file 2: Figure S4, *p* value < 2.2e-16, Χ^2^ test, Additional file 1: Table S3). Furthermore, epigenetic marks that are usually associated with actively transcribed promoters—H3K4me1, H3K27ac, and H3K4me3—were present in the majority of promoters of novel transcripts and enriched compared to the rest of the genome (Fig. 2c, Additional file S2: Figure S3f, g). In addition, 53.4% of captured transcripts overlapped H3K36me3 broad peaks from liver tissue (45.7% for lowly expressed, 62.2% for intermediate, and 96.1% for high), an overlap which is expected due to their spliced nature. Despite the previously described overlaps with CAGE and epigenetic marks, it should be noted that the CaptureSeq methodology is still limited by its short-read sequencing component in precisely defining transcript margins and would require further validation.

To further demonstrate the existence and structure of the captured transcripts, we selected 30 at random, successfully validating 90% of transcripts and 89% of their splice junctions (Additional file 2: Figure S5, Additional file 1: Table S4). Taken together, we find that the majority of our captured transcripts are novel and are statistically significantly enriched for some properties of active non-coding RNAs.

### Functional relevance of captured transcripts and their genomic regions

The challenge of functionally annotating captured transcripts has been addressed with in silico analyses of tissue-specific expression, enrichment for known functional elements, and GWAS SNPs, as well as through individual cases of ten independently functionally validated lncRNAs.

**Fig. 3:**
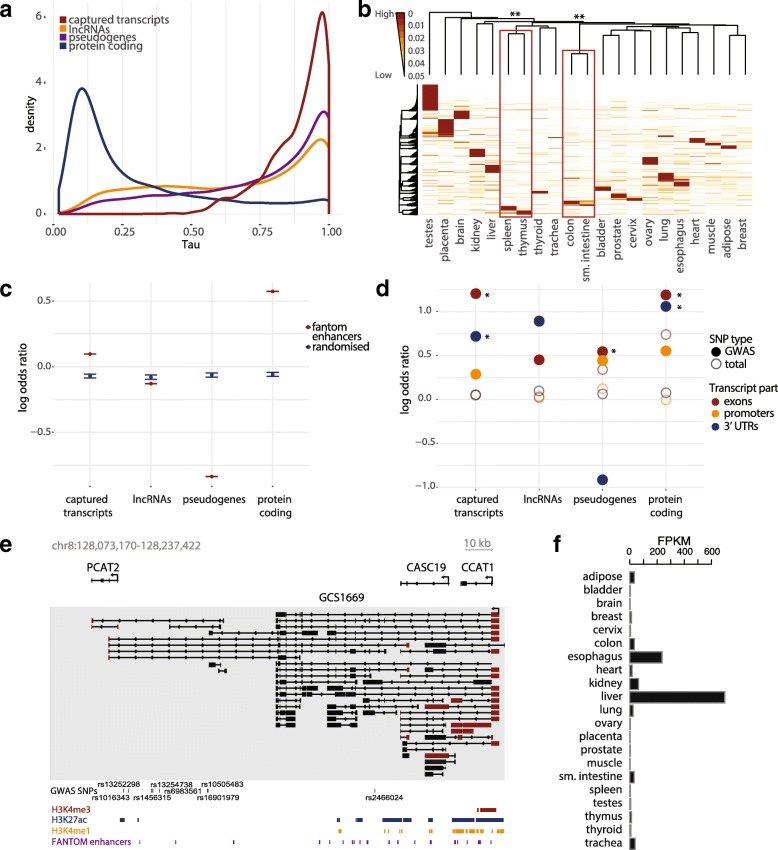
Functional properties of captured transcripts. **a** Comparison of tissue-specific expression of captured transcripts to lncRNAs, pseudogenes, and protein-coding genes (Illumina Body Map), as measured by Tau tissue specificity index (0 for broadly expressed, 1 for tissue specific genes) [79]. **b** *Heatmap* of tissue-specific captured transcripts (τ > 0.8) across tissues. Unsupervised clustering performed on τ components (1-Expression/max(Expression)), colored by tissue specificity from low (*white*) to high (*red*). Statistically significantly non-randomly clustered branches after 10,000 bootstraps, as calculated by Pvclust [115] are marked by *red rectangles*. **p value of a cluster branch < 10^–3^. **c** Enrichment of genomic regions of captured transcripts for known FANTOM enhancers. Log odds ratios (ORs) of enrichment (with 95% confidence intervals) compared to lncRNAs, pseudogenes, and protein-coding genes. Genomic regions of both introns and exons were included in the analysis. FANTOM enhancers in *red*, randomized regions in *blue*. **d** Enrichment of GWAS SNPs in transcript regions. Log OR of enrichment for GWAS SNPs (*p* value < 5 × 10-8), compared to intronic regions. Exons in *red*, promoters in *yellow*, 3’ UTRs in *blue. Hollow circles* denote enrichment for common SNPs. Statistically significant adjusted *p* values (Χ^2^ test, *p* values < 0.05) are denoted with *asterisks*. **e** Example of a captured transcript with independently validated function. Transcript GCS1669 overlaps three known lncRNAs, with CCAT1 being functionally validated in liver and prostate carcinogenesis. *Gray box* marks captured region. Previously observed splice sites are denoted in *red*. **f** Expression levels of transcript GCS1669 across tissues

First, we investigated whether the novel transcripts were expressed in a tissue-specific manner similar to other lncRNAs, which signifies potential importance in programming and behavior of cell lineages [1, 3]. We calculated Tau index (τ) to detect condition-specific profiles of the captured transcripts [79]. The majority of transcripts (81%) presented a tissue-specific profile (τ > 0.80), mostly from known transcriptionally diverse tissues including testes and brain, but also placenta, emphasizing the dynamic and divergent nature of the placental transcriptome (Fig. 3a, b) [1, 3, 5]. A small subset of tissues that are functionally related—colon and small intestine, spleen and thymus—formed statistically significant clusters (*p* values < 10^–3^, Pvclust multiscale bootstrap resampling, Fig. 3b), while randomized expression across conditions tissues presented no significant correlations between samples (Additional file 2: Figure S6). Finally, a number of captured transcripts were also overexpressed in tissues that are relevant for individual diseases (Additional file 1: Table S3).

Second, we examined the possible mode of action of captured transcripts by determining their overlap with functionally annotated genomic regions. About one-third of captured transcripts (36.5%) overlapped previously reported transcribed enhancers (eRNAs) from the FANTOM project, for which they were enriched compared to the genomic average and randomized locations (Fig. 3c) [80]. A similar enrichment was obtained by analysis of ChromHMM genome segmentation, representing chromatin states defined by combinations of multiple epigenetic marks. Of captured transcripts, 85.8% overlapped enhancer regions and showed enrichment for loci associated with weak active enhancers (Additional file 2: Figure S7) [12]. However, 95% of eRNAs are single-exonic [80], while we report only multi-exonic transcripts. Enhancer RNAs, whether polyadenylated or not, can be unidirectionally transcribed from enhancer regions (1D eRNAs) or more commonly in a bidirectional manner [81]. Only one-third of captured transcripts (34%, see “Methods”) came from bidirectional promoters, though bidirectionality was more prevalent in our transcripts that overlap FANTOM enhancers (48%). These results imply that even though a significant proportion of captured transcripts could theoretically have a role as eRNAs, for which further functional validations are required, the potential function of the remainder could encompass the diverse repertoire of mechanisms available to other types of lncRNAs [82].

Third, we calculated the proportion of bases with GWAS SNPs in different regions of the captured transcripts, since it has previously been observed that the polygenic effects of SNPs in GWAS studies are enriched for those associated with exons and regulatory regions [18, 48]. Even though tag SNPs are not causative, those that overlap functional regions explain more variance and are more likely to be associated with a phenotype than others. We observed enrichment of the disease-associated variation in promoters, exons, and 3’ UTRs compared to introns of captured transcripts, comparable to that in lncRNAs and protein-coding genes (Fig. 3d). Out of 1775 transcribed loci, 415 (23%) contain a GWAS SNP, 166 (9.2%) in their exons. We further investigated whether transcripts contain a previously established expression quantitative trait locus (eQTL) SNP from the GTEx study [54] and we observed such overlap in 83 cases, 55 of which were in exonic regions. We provide several examples of captured transcripts with exonic eQTLs that influence expression of protein-coding genes implicated in the phenotype associated with the captured transcript’s haploblock of origin (Table 1). In addition, utilizing our melanoma samples (see below), 152 transcripts exhibit allelic imbalance, showing significant difference in expression in relation to the SNP variants they contain (FDR < 0.1). The similar patterns of disease-associated variation in known genes and our novel transcripts, along with the presence of eQTL SNPs and allelic expression changes in response to genetic variation, supports their functional relevance and suggests some may play a role in complex human traits and diseases.

**Table 1:** Examples of captured transcripts with exonic eQTLs. Protein-coding genes whose expression is influenced by eQTLs are characterized by their function and tissue expression in GTEx. In brackets: fold change overexpression of associated genes in specific tissues compared to their average expression, as given by GTEx or Human Integrated Protein Expression Database (HIPED) [118] in case of KALRN

Captured transcript	Highest tissue expression	Haploblock associated phenotype	eQTL	Associated gene	Gene function	Tissue expression
GCS0300	Cervix	Prostate cancer	rs72928357	MYEOV	Stimulation of cancer growth and proliferation [119]	Vagina (2.6x)
GCS0406	Heart	HDL cholesterol	rs7134375	PDE3A	Hypertension, fat metabolism [120]	Heart (19x)
GCS0736	Liver, thyroid	HDL cholesterol	rs11875196	LIPG	Modulation of HDL cholesterol [121]	Liver (14x), thyroid (78x)
GCS1080	Heart	Mean platelet volume	rs13058993	KALRN	Activates Rho GTPases to regulate actin cytoskeleton [122]	Platelets (10x, HIPED), heart (2x)
GCS1212	Thyroid	Thyroid function	rs4835532	Mineralocorticoid receptor (NR3C2)	Regulation of cellular ion concentrations [123]	Thyroid (7.0x)
GCS0965	Testes	Age at first menstruation	rs708984	PCSK2	Conversion of proinsulin to insulin [124]	Testis (2x), thyroid (15x)
GCS1190	Kidney	Metabolic traits in urine	rs2348209	ENPEP	Peptide cleavage [125]	Kidney (11x)

Finally, we report ten captured transcripts that have been independently functionally annotated after the design of our experiment based on GENCODE v.12 (Fig. 3e, f, Additional file 1: Table S5, Additional file 2: Figure S8), including two transcripts that were identified through CaptureSeq technology. Captured transcript GCS1669 contains most of the splice sites of three independently reported lncRNAs—CCAT1, CASC19, and PCAT2—in addition to multiple novel exons and isoforms that encompass all three (Fig. 3e). Even though it was first reported in colorectal cancer, CCAT1 is involved in multiple malignancies based on its enhancer regulation of MYC [83, 84]. Interestingly, GCS1669 is specifically expressed in liver, while CCAT1 has been shown to promote hepatocellular carcinoma [84]. Other examples include GCS1684 that overlaps the lncRNA CCDC26 in a haploblock associated with growth of white blood cells. While CCDC26 controls myeloid leukemia cell growth [85], GCS1684 is specifically expressed in spleen, a major storage location for leukocytes (Additional file 2: Figure S8i) and shows significant allelic imbalance in 8/13 melanoma samples (FDR < 0.05). Similarly, GCS0593 is specifically expressed in thyroid tissue, comes from haploblock associated with thyroid hormone levels and thyroid cancer, while overlapping lncRNA GCS0586 that causes proliferation of thyroid carcinoma, likely through Wnt signaling pathway [86]. In addition, two non-coding transcripts, CUPID1 and CUPID2, have been identified with CaptureSeq technology, functionally validated with RNA-seq, HiC, and knockout experiments, and have been implicated in modulating DNA repair in breast cancer [87].

### Identification of novel transcripts implicated in cutaneous melanoma

**Fig. 4:**
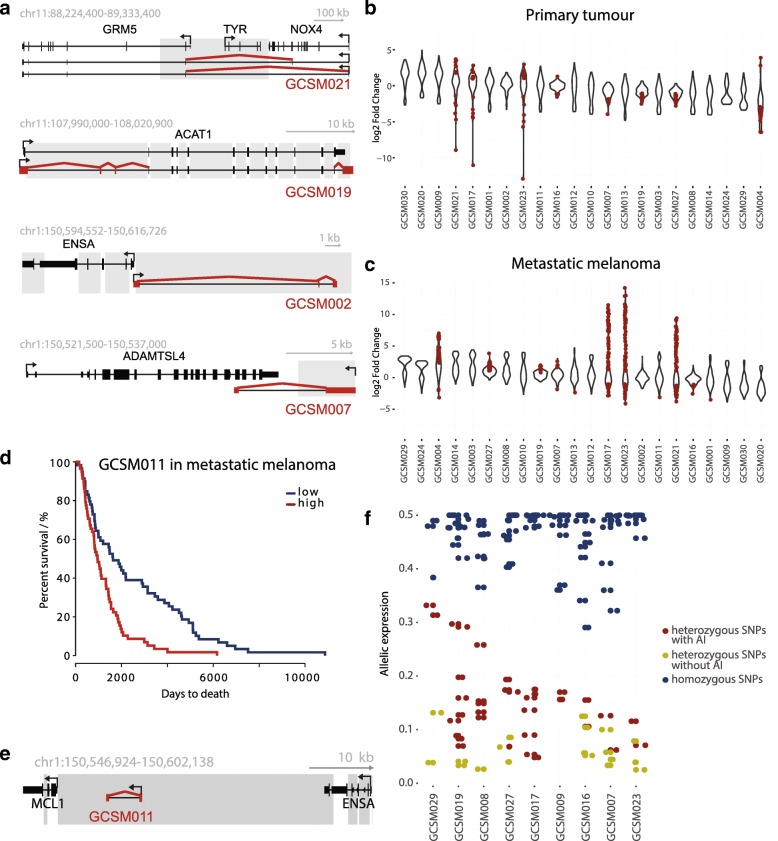
Identification of novel transcripts expressed in skin cutaneous melanoma. **a** of novel transcript types identified through CaptureSeq on 13 melanoma transcriptomes, targeting regions proximal to key melanoma genes. *Red lines* denote novel splice junctions, *red blocks* novel exons, and *gray boxes* the captured regions. From *top* to *bottom*: a fusion protein between GRM5 and NOX4, novel exons on ACAT1, novel lncRNAs bidirectional to ENSA, and an antisense lncRNAs overlapping ADAMTLS4. **b**, **c** *Violin plot* of fold change of novel transcripts in primary tumor and metastatic samples vs normal. *Red dots* denote significant differences (FDR < 0.01). Out of 31 novel transcripts, 22 were detectable in both TCGA primary tumors and metastatic samples. **d** *Kaplan–Meier survival curve* for captured transcript GCSM011 in metastatic melanomas. *Red lines* mark the groups in the upper half of transcript expression and *blue* for the lower half. **e** *Schematic representation* of genomic loci of GCSM011 between MCL1 and ENSA. **f** Allelic imbalance of captured transcripts in haploblocks associated with melanoma. Heterozygous sites were predicted with QuASAR [116] and allelic imbalance calculated with MBASED [117]. *Y-axis* represents median allelic expression across heterozygous SNPs. Allelic imbalance displayed as absolute value of 0.5 – allelic imbalance. Homozygous and heterozygous SNPs with allelic or without allelic imbalance are shown in *blue*, *red*, and *yellow*, respectively. At least 30 reads had to be observed over a SNP, with significance cutoff of FDR < 0.1

We investigated the utility of the CaptureSeq approach on genomic regions associated with disease pathology with application to melanoma. We performed CaptureSeq on the transcriptomes of 13 skin cutaneous melanoma samples, targeting nine additional haploblocks with melanoma susceptibility GWAS SNPs (Additional file 1: Tables S1h, i). Vicinity to the previously annotated genes allowed us to identify a diverse set of interactions between the 31 novel transcripts and the known genes relevant to melanoma, such as fusion transcripts (e.g. CUL5-ACAT1, NOX4-GRM5), novel exons (e.g. ACAT1, TYR, ARNT1, MCL1), bidirectional transcription from the same promoter (e.g. ENSA), and antisense lncRNAs (e.g. ADAMTSL4) (Fig. 4a, Additional file 1: Table S6). We validated our transcripts selectively by polymerase chain reaction (PCR) and sequencing (80% validation rate, Additional file 2: Figure S9) and globally with data from The Cancer Genome Atlas (TCGA) for melanoma tumors and metastases [22, 24, 26] where one-third of transcripts—nine from primary tumors and eight from metastases—were present at FPKM > 1 in at least three samples even without CaptureSeq enrichment. These novel transcripts and exons were differentially expressed (FDR < 0.01) in melanoma primary tumors and metastases compared to normal in 36% and 50% percent of samples, respectively (Fig. 4b) and five of them contained exonic eQTL SNPS identified by the GTEx consortium (GCSM002, GCSM004, GCSM0019, GCSM0026, GCSM0028). For example, GCSM0019 contains eQTLs rs11212525 and rs9666209 that are associated with expression of ACAT1, regulator of antitumor response of CD8(+) T-cells [88] as well as expression of angiogenesis mediator ATM [89]. In addition, transcript GCSM011, which is located near the known oncogene MCL1 [27], was associated with significantly decreased survival rate (*p* value 0.0002, Χ^2^ test, FDR < 0.005), marked by a 25% decrease in survival after five years with metastatic melanoma (Fig. 4d, e). As expected for melanomas [90], a high proportion of transcripts (29%) showed allelic imbalance, with the significantly different expression of transcripts depending on the allelic origin (Fig. 4f). In summary, diversity of melanoma transcripts captured from regions associated with cutaneous melanoma presents the potential of CaptureSeq to provide high-resolution patient-specific information on well-described genomic loci related to various diseases.

### Database of novel transcripts

Our approach allowed us to build an easily accessible resource of novel disease-associated transcripts, available online at http://gwas-captureseq.dingerlab.org for interactive examination and visualization. The resource integrates the genomic locations of novel transcripts with raw experimental data, transcript models and their expression, as well multiple layers of publicly available data from epigenetic markers to eQTL-associated variation.

## Discussion

Here, we have presented the first targeted assessment of transcriptional potential for all known intronic and intergenic haplotype blocks associated with complex traits. Even though we examined only 2% of the genome, conservatively focusing on only multi-exonic transcripts, the higher resolution of the CaptureSeq approach in combination with information from 21 tissues increased the number of observed lncRNA genes in GENCODE (v.19) by 13% and resulted in the discovery of hundreds of novel transcripts, isoforms, and exons that come from regions associated with diseases, 70% of which were not previously detected. In concordance with the previous observation that 76.6% of all intergenic GWAS SNPs lie within DNAseI hypersensitive sites that are functionally related to transcriptional activity [32], we observed transcriptional events from 85% of the captured intergenic regions, using only a select number of tissues and conservative cutoffs (Fig. 1c).

The comprehensive computational analysis of novel transcripts indicated similarity to other actively transcribed non-coding RNAs. The captured transcripts were mostly non-coding and non-conserved, while enriched for characteristic epigenetic marks of active transcription (Fig. 2, Additional file 2: Figure S3). The large overlap with previously observed enhancers (37%) suggests enrichment of the novel transcripts for enhancer RNAs, a set of regulatory transcripts that are characterized by low expression, tissue specificity, redundancy in promoter regulation, but also enrichment in variation [35, 43]. The current view in the field is that while some eRNA transcripts are likely non-specific noise, the expression of others correlates with higher binding of transcriptional co-activators, greater chromatin accessibility, and formation of enhancer-promoter loops [91] and there is evidence that disruption of their sequences leads to dysfunctional enhancer activity [92]. On the other hand, the majority of captured transcripts (63%) do not overlap with previously reported enhancers from the FANTOM 5 project, do not show other typical eRNA properties (bidirectionally expressed in only 25% of cases, multi-exonic) and therefore likely have a different mode of function.

We provide multiple lines of evidence that the new transcripts are not simply spurious readouts of the genome by RNA polymerase II. The transcripts were depleted in gene deserts and introns and enriched in known exons, while presenting a non-uniform distribution across the targeted haploblocks. While non-conserved, they were enriched for epigenetic markers of active transcription and showed tissue specificity and enrichment for weak active enhancers. Furthermore, captured transcripts contained exonic GWAS SNPs and eQTLs, with multiple examples of allelic imbalance in melanoma samples, providing further evidence for importance of disease-associated variation in the function of captured transcripts.

Non-coding transcripts are often expressed at orders of magnitude lower levels than protein-coding genes and therefore considered less likely to be of functional relevance. The lower overall abundance of lncRNAs is, at least partly, due to heightened spatiotemporal precision in their expression: some, such as oncogene HOTTIP [93], are present in only a small proportion of cells, some are distributed in a highly precise pattern across tissues [94], while others are present in a short timeframe through bursts of expression [95]. Furthermore, single-cell RNA-Seq studies have shown that rare cell types may be represented by just a few cells within a community of hundreds or thousands of cells [96] and regulatory molecules that establish their identity will necessarily be represented at low overall abundance. Therefore, defining the repertoire of non-coding RNA regulators that are used by the cells, though confounded by transcriptional noise and random readouts of RNA polymerase II, should be based on multiple lines of evidence and not expression levels alone.

CaptureSeq allows a reversal of the usual approach of discovering novel transcripts and investigating their expression from genome regions with indices of function, by targeting regions with known but unexplained function and investigating if they are transcriptionally active. We therefore expect our novel transcripts to be enriched for functional non-coding RNAs. Though it would be impractical to functionally validate all of the identified transcripts, some have already been independently functionally validated and demonstrate the great potential of the dataset. In addition to the eight examples that were reported in the literature, Betts et al. provided evidence that one of the transcripts we identified at the 11q13 breast cancer risk locus, named CUPID2, alters breast cancer risk by modulating the DNA damage response [87].

Additionally, by enriching 13 melanoma transcriptomes for intergenic and intronic loci that are associated with risk for melanoma, we identified a number of novel biomarkers and potential regulators, even in the previously well-characterized melanoma transcriptome [45, 47]. We discovered fusion transcripts of key melanoma genes, multiple novel exons as well as a number of previously unreported lncRNAs whose presence correlates with clinical outcomes. Many of these transcripts were detectable in the TCGA datasets, but despite differential expression in cancer compared to normal samples had remained uncharacterized due to the limits of the reference gene annotation.

Our results demonstrate that we have only just started to understand the transcriptome, the complexity of which may have a profound impact on human development and human health. They further point to the crucial importance of high-resolution technologies such as CaptureSeq to eliminate biases resulting from abundantly expressed transcripts. We expect that the provided freely available database of novel transcripts adds to our understanding of the human genome and will serve as an important resource in the study of complex diseases.

## Methods

### Cell and tissue samples

Normal human tissue RNA was obtained from the FirstChoice Tissue Panel (Ambion AM6000) and Human Breast Total RNA (Ambion AM6952). A wide variety of tissues (21) were investigated to ensure that our results regarding the number of expressed intergenic regions and the tissue-restricted nature of transcripts had low susceptibility to false negatives. Melanoma RNA was obtained from patient cell lines, including three originating from metastasized stage IV melanoma (A-series) and ten originating from resected lymph nodes from patients with stage 3 disease (C-series) [97, 98]. Cell line authentication using short tandem repeats (STRs) confirmed each as being from a single source and matching the patient germline. Cell lines have been confirmed negative for Mycoplasma using the MycoAlert mycoplasma detection kit (Lonza). Cell lines were established at the QIMR Berghofer Medical Research Institute, as described previously [97, 98], with informed patient consent under protocols approved by the QIMR Berghofer Medical Research Institute Human Research Ethics Committee (HREC/14/QPAH/495). Breast cancer and RNA was extracted with RNeasy columns (Qiagen). All samples are listed in Additional file 1: Table S8.

### Design of pilot GWAS capture array

The pilot capture array was designed to target intergenic linkage disequilibrium (LD) blocks surrounding disease-linked SNPs from the NHGRI catalog of GWAS [23]. LD blocks around intergenic GWAS SNPs were estimated as previously described [99]. All GWAS catalog SNPs (with *p* values < 1e^-5^) were utilized. LD blocks were calculated by identifying the most distant 3’ and 5’ SNPs with an r^2^ > 0.5 (using HapMap SNPs release 22 from the CEU population [100]) then extending the block to the nearest recombination hotspots [101]. Total LD block size was restricted to 1 Mb. LD blocks without RefSeq genes were considered as candidate intergenic LD blocks. The pilot capture targeted a total of 339 separate LD regions. Five housekeeping genes (GUSB, HPRT1, HMBS, TFRC, TBP) were also included as positive controls for gene detection.

### Library preparation and capture sequencing for pilot experiment

Pilot capture sequencing was performed similar to previously described [46] by combining and modifying the NimbleGen SeqCap EZ Library SR User’s Guide V3.0 and the NimbleGen Arrays User’s Guide: Sequence Capture Array Delivery v3.2. Three micrograms of total RNA from 20 human tissues (FirstChoice Tissue Panel [Ambion]) were pooled together and ribodepleted in 5-μg batches (Ribo-Zero™ [Epicentre]) before being pooled again. Sequencing libraries were made with 400 ng of ribo-depleted RNA using the Illumina TruSeq® Sample Preparation Kit (unstranded), all libraries utilized adaptor sequence 12. One-twentieth of completed but unamplified library was amplified according to the Illumina “enrich DNA fragments” method and analyzed by the Bioanalyser to validate correct library construction. The remaining sample from six libraries was pooled and amplified according to the Nimblegen Pre-capture LM-PCR specifications, with the modification of ten cycles of amplification. Input into the capture hybridization was 1ug of library. Capture hybridization was performed as previously described [46], with the following modifications. After drying down in a vacuum concentrator, the samples were resuspended in 9.2 μL of nuclease and nucleic acid-free water. Hybridization enhancing (HE) oligonucleotides used were 1 μL of 1000 μm TS-INV-HE Oligo 12 and TS-HE Universal Oligo 1 and were added after the sample was solubilized at 70 °C for 10 min. Pre-capture and post-capture samples (both a pool of 21 human tissues) were each sequenced on a single lane of an Illumina® HiSeq.

### Design of GWAS capture pools

Oligonucleotide probes were designed to capture all GWAS loci LD blocks that did not contain a coding exon using a Roche NimbleGen SeqCap EZ Choice XL Library. All intergenic or intronic disease-linked SNPs from the NHGRI catalog of GWAS [23, 44] (accessed 30 July 2012) were downloaded and filtered to retain only those with *p* values < 5e^–8^. LD blocks were calculated with a two-step process. First, SNAP [102] was used to extend GWAS LD blocks from the GWAS SNP to the furthest SNPs with an r^2^ > 0.5 using Hapmap23 and 1000 Genomes SNPs [103]. LD block size was limited by a 500-kb cutoff up and downstream of the GWAS SNP. Next, in cases where there were insufficient SNPs to define an LD block; specifically, when there were no SNPs with an r^2^ < 0.6 on one or both sides of a GWAS SNP, the side(s) of the LD block with insufficient SNPs was extended to the nearest recombination hotspot [101]. Any LD blocks containing GENCODE V12 or RefSeq coding exons or under 3 kb in total were excluded, leaving 296 totally intronic or intergenic GWAS blocks.

To ensure continuity between the pilot array and the GWAS capture pools, all exonic regions from multi-exonic transcripts identified in the pilot array capture sequencing were included as probe targets in the updated design (Additional file 1: Table S1e). Control intronic regions from pilot capture sequence transcripts were tiled to assist in differentiating exons from introns. A 200 nt to 1 kb region from one random intron per expressed loci was selected. Any intronic targets with a repeat content > 75% were filtered out and another intron randomly picked from the locus (if possible) (Additional file 1: Table S1f).

Other control sequences in the design included five housekeeping genes (GUSB, HPRT1, HMBS, TFRC, TBP), six single-exon transcripts plus up to 1 kb of upstream and downstream genomic sequence, a gene desert region, and probes to the ERCC Spike-In Control set (Life Technologies) (Additional file 1: Table S1g).

The design also included genomic loci associated with breast cancer and melanoma. Breast cancer regions were selected heuristically, while for melanoma LD blocks were defined from a SNP as per the GWAS LD blocks (above). Loci were filtered to remove the exons (plus 100 nt on each side) of highly expressed protein-coding genes, as well as any target region under 200 nt created by this step. The remaining coding genes, intergenic and intronic regions from each genome loci were included in the design.

Design of probes from target regions and probe synthesis was performed by Roche NimbleGen. Probes were allowed a maximum of five matches to the human genome. Synthesized probes covered 80.4% of target regions directly, with 90.6% of target regions estimated as being available for the capture protocol.

### Library preparation and capture sequencing

Libraries were prepared and capture hybridizations were performed as previously described [64] on RNA from 21 tissues and 13 melanoma samples. Briefly, RNA was DNAse-treated with TurboDNase (Life Technologies), confirmed DNA-free, RNA integrity was confirmed by Agilent 2100 Bioanalyzer (Agilent Technologies). rRNA depletion (Ribo-Zero™ [Epicentre]) was performed on 5 μg of total RNA. ERCC RNA Spike-In Control mix 1 or mix 2 (Life Technologies) were added to ribodepleted RNA to a final dilution of 1/100. Libraries were prepared with the TruSeq Stranded mRNA Sample Preparation Kit (Illumina) and 9–12 cycles of pre-capture LM-PCR performed on tissue samples as required. All Melanoma samples were amplified with the same number (10) of pre-capture LM-PCR cycles to prevent differences between the samples due to PCR biases. One sample was excluded at this point as it had a poor yield that required extra cycles of pre-capture LM-PCR, leaving 13 samples remaining. Multiplex library pools were created by mixing equal amounts of five pre-capture sample libraries and capture hybridization performed on 1 μg of the pooled library. Melanoma A-series samples were each randomly assigned to one of three capture hybridizations to ensure any expression differences between the A and C-series samples were not due to an A-series batch effect.

Post-capture LMPCR was performed for 17 cycles. One or two multiple library pools (representing five or ten original libraries) were sequenced per lane on an Illumina HiSeq, paired-end sequencing of 100 nt reads.

### Enrichment quantitative PCR (qPCR)

Enrichment qPCR was performed as previously described [64] using Sybr Green PCR Master Mix and real-time cyclers (Applied Biosystems). Successful capture was confirmed by enrichment of Roche capture controls and transcripts specifically targeted by the design, while capture specificity was confirmed by depletion of negative control transcript not targeted by the capture. A minimum average enrichment of 50-fold (as determined by qPCR) was required for capture hybridizations to be deemed successful. Any capture hybridizations with average enrichment under this threshold were repeated.

### Definition of the capture space

The 339 haploblocks from the pilot study (Additional file 1: Table S1b) and the additional 296 haploblocks with GWAS SNPs of p < 5e^–8^ were collapsed in R (v.3.1.0) into 561 genomic regions (Additional file 1: Table S1a) from which we eliminated all GENCODE (v.12) exons with gene type “protein_coding” or “lincRNA” as well as pilot introns that serve as a negative control (Additional file 1: Tables S1b, e–g). Similarly, nine haploblocks containing SNPs associated with melanoma (Additional file 1: Table S1h) were cleaned of known exons (Additional file 1: Table S1i).

### De novo transcript assembly and quantification

The sequenced sample libraries were trimmed with Trim Galore (v.0.2.8) and assembled with Trinity (v.20140710beta) [104] for each tissue or melanoma sample. After mapping the transcripts back to the hg19 genome with GMAP (v.2014-02-28) [105], the transcripts were merged independently for primary tissues and melanoma samples with Cuffmerge (v.1.0.0). A fasta file of transcripts was created with gffread function from Cufflinks (v.2.2.1) [106], the read libraries were then mapped to the tissue and melanoma transcriptomes with STAR (v.2.4.0d) [107], and counted with RSEM (v.1.2.12) [108]. The counts reported with RSEM were normalized with R package DESeq [109] based on the batch (Sup. Table S8) with method “blind” and sharingMode “fit-only.” Only transcripts that overlapped the Capture Space with RKPM > 1 in at least one tissue and the isoforms that contributed > 1% were reported. The transcripts are located in Additional file 1: Table S2. The samples from the breast cancer cell lines were trimmed with Trim Galore (v.0.2.8) and assembled using Cufflinks (v.2.2.1) [106].

### Transcript characterization

Overlap with the annotated transcripts for Fig. 2a was calculated with function “findOverlaps” and width of sequence overlap with function “pintersect” in R. The coding potential of the transcripts was assigned with the Coding-Potential Assessment Tool (CPAT) (v.0.9) [72] and CPC [73]. Conservation scores were assessed with Bioconductor package *phastCons100way.UCSC.hg19*. In order to define tissue specificity of the transcripts, we employed Tau tissue-specificity index after vst transformation of the count data [110]. Assessment of bidirectional transcription was performed on captured transcripts by counting the number their promoters overlapped, once their genomic sequences were extended at 5’ ends by 500 bp.

### Enrichment analyses

Log odds ratio (OR) is calculated from contingency table of Fisher’s exact test for overlap between a ROI and annotation whose enrichment is being tested. More specifically, if we are looking at a region R (either transcript or promoter loci) and overlap with annotation A (GWAS SNPs, epigenetic marks, etc.), then *a* is the number of nucleotides of overlap between R and A, b is the number of nucleotides in annotation A without *a*, *c* is the number of nucleotides in R that do not contain A, and *d* is the total number of genomic nucleotides without R and A. The OR is then given as (*a/b*)*/*(*c/d*). Confidence intervals for log OR are then calculated as 1.96 times standard error, which is given as a square root of 1/*a* + 1/*b* + 1/*c* + 1/*d*. The *p* value of the enrichment is calculated through Chi-square test and R function *chiseq.test*. For the enhancer enrichment analysis in Fig. 3c, we excluded genomic regions that were in the 500-bp proximity of GENCODE promoters and that contained GENCODE exons and 200 bp around them. For Additional file 2: Figure S4 and CAGE mark enrichment, the length of genome required for this calculation was reduced by the regions overlapping promoters and exons of GENCODE genes, and the regions that were analyzed for enrichment contained 5’ of captured of transcripts + – 500 bp. Negative controls were defined with R package ChIPseeker, with 100 randomizations of whole captured transcript genomic start sites expanded to the width of + – 500 bp. GWAS enrichment analysis for transcript elements (promoters, exons, 3’UTRs) in Fig. 3 was calculated in relation to intronic content of GWAS and total SNPs. The same number of elements was used in the analysis for lncRNAs, pseudogenes, and protein-coding genes, identical to the ones in captured transcripts. *P* values were calculated from Χ^2^ test, with alternative “greater than.”

### Calculation of normalized counts for aggregate plots

Expression of captured transcripts was determined based on the Illumina Body Map libraries for testis and liver, using the STAR genome aligner (v.2.4.0d) and RSEM (v.1.2.12). Genomic regions of 5’ ends of captured transcripts that were detectable in a tissue (FPKM > 0), were overlapped with CAGE tags (FANTOM5) and epigenetic marks for the appropriate tissues in the area of + – 5 kb. The counts were normalized by the number of transcripts. Negative control regions were calculated with R package ChIPseeker for the same number of transcripts as the visualized captured transcripts and expanded to the width of 10 kb [111]. Known promoters and exons of GENCODE genes were excluded from these areas, that were then overlapped with CAGE and epigenetic marks, as described above. Positive control was based on lncRNAs with median expression over Illumina Body map tissues matched to median of captured transcripts in the same libraries.

### Analysis of melanoma samples

The transcripts were assembled and quantified as described previously. The annotation was performed with Cuffcompare (v.2.2.1) [112]. Overlap with the TCGA melanoma samples for primary tumors, metastases, and normals were calculated in R v.3.2.0, while eliminating counts from any previously annotated GENCODE exons. Kaplan–Meier curves based on splitting the populations into half to highly and lowly expressed transcripts were plotted with R package *survival*.

### Transcript validation

Thirty transcripts, ten from each of the low, medium, and high expression categories, were chosen at random to validate transcription from 30 loci. Primers were designed using Primer3 to amplify across splice junctions to prevent false positive detection due to DNA contamination. Primers sequences are available in Additional file 1: Table S4. Acceptable FirstChoice Tissue Panel (Ambion AM6000) RNA quality and quantity was determined by Agilent Bioanalyser 7900 Picochip and Qubit 2.0 Broad Range Assay (ThermoFisher). Reverse transcription was performed on 70–600 ng of the sample RNA using the SuperScript IV Reverse Transcription Kit (Invitrogen) with random hexamers, according to standard protocol. PCR amplification of targeted transcripts was performed on 5–100 ng of complementary DNA using Phusion High-Fidelity PCR Master Mix with HF® Buffer. An initial denaturation step of 30 s at 98 °C was performed followed by 30–37 cycles of 10 s at 98 °C, 30 s at 45–65 °C, and 15 s at 72 °C, with a final extension of 5–10 min at 72 °C. The samples were run at 75 V for 1.5 h on a 1.7% agarose gel containing GelGreen Nucleic Acid Gel Stain (Biotium). Bands of the correct amplicon size were excised from the gel and the DNA purified using the QIAquick Gel Extraction Kit (QIAGEN). The DNA samples were submitted to the Garvan Molecular Genetics facility for Sanger sequencing. The resulting sequences were aligned against the sequences of the targeted transcripts with BLAST [113] and those with at least 95% homology were reported as a match. Tissue-specific isoform sequences are available in the source data files, as described below.

For melanoma samples, a similar procedure was performed against five transcripts, with primers described in Additional file 1: Table S7. PCR products were cleaned using Agencourt AMPure® beads (Agilent), followed by Sanger sequencing using BigDye® Terminator v3.1 (Applied Biosystems), which was cleaned by Agencourt CleanSeq® beads (Agilent). The cleaned sequencing product was run on a 3130 × l 16 capillary genetic analyzer (Applied Biosystems) and the results were analyzed using Sequence Scanner v2 (Applied Biosystems).

## Additional files

Additional file 1: Supplementary **Tables S1** to **S3** provide genomic regions and annotation for captured regions and transcripts. **Tables S4** and **S4** provide information on PCR validation and independent functional validation, respectively. **Tables S6** and **S7** provide genomic information on captured regions and transcripts specific for melanoma. **Table S8** provides information on used samples. (XLS 18124 kb)

Additional file 2: Supplementary figures and **Tables S1** to **S9**. (DOCX 4403 kb)
