# Supplementary material for: Intergenic disease-associated regions are abundant in novel transcripts
Source: Genome Biol. 2017 Dec 28;18:241. doi: 10.1186/s13059-017-1363-3 (PMC5747244; doi:10.1186/s13059-017-1363-3)
Supplement: Supplementary file 2 — Supplementary figures and Tables S1 to S9. (DOCX 4403 kb) [file 13059_2017_1363_MOESM2_ESM.docx]

# Supplementary Figures

Figure S1. Quality control of genomic loci with captured transcripts. a. Enrichment of single-exonic (blue) and multi-exonic (red) transcripts in control regions: gene desert (chr7:41189784-41589784), intronic and exonic regions. “Exon proximal” represents intronic regions that are proximal (<200bp) to exons. Y axis represents natural log of odds ratio that a nucleotide from a captured region contains an assembled transcript, compared to the total of captured genomic loci. Horizontal lines depict confidence intervals, defined as 1.96*standard error (see Methods). b. Percentage overlap of control captured regions from panel a. with single and multi-exonic transcripts. c. Testing the hypothesis that transcripts are random RNA polymerase II readouts by assessing the relationship between haploblock length and transcript expression. Under transcriptional noise scenario, haploblocks without transcripts should be shorter: since we report the maximum expression of a transcript per haploblock, in the scenario of random transcription it would be expected that longer haploblocks have higher odds of randomly gaining a highly-expressed transcript. No such bias was observed in the distribution of the multi-exonic transcripts across haploblocks, and haploblocks without novel transcripts were not shorter on average (p-value 0.40). Furthermore, we randomized genomic loci of multi-exonic transcripts and examined the properties of empty haploblocks. In this simulation, empty haploblocks were significantly shorter (p-value< 2.2x10-16).


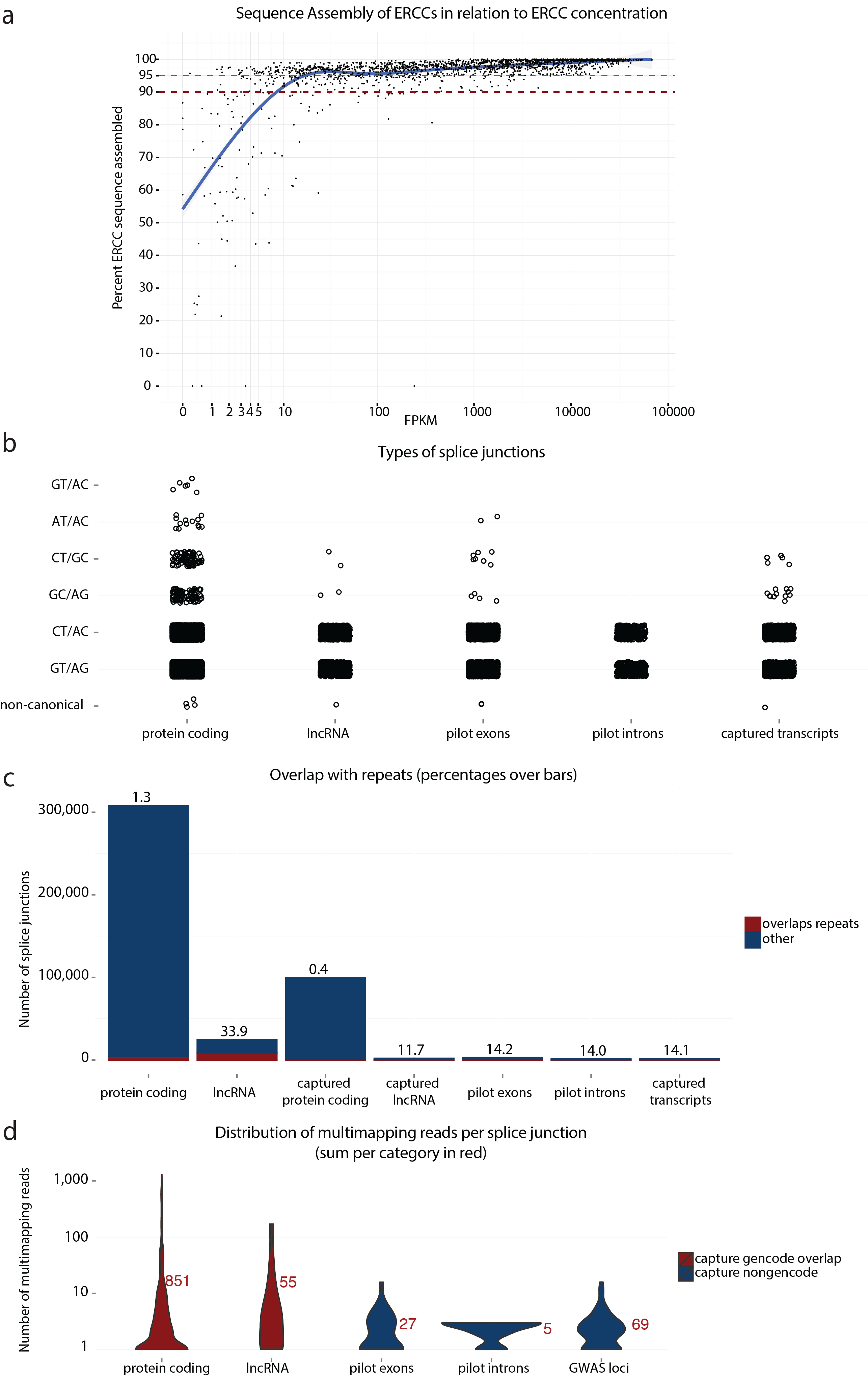


Figure S2. Quality control of captured transcripts. a. Quality of sequence assembly in relation to ERCC concentration. ERCC spike-ins were added to the samples, and the quality of their assembly was measured based on their concentration. b. Types of splice junctions observed by STAR aligner for the de novo assembled multiexonic transcripts overlapping pilot exons, pilot introns or loci with GWAS SNPs, compared to canonical protein-coding or non-coding genes from GENCODE v19. c. Overlap of assembled transcripts with repeats, compared to GENCODE annotation. Percentage of overlap with repeats over the bars. d. Control for multimapping reads in splice junctions of captured transcripts, compared to GENCODE annotation. Sum of the reads per category in red.


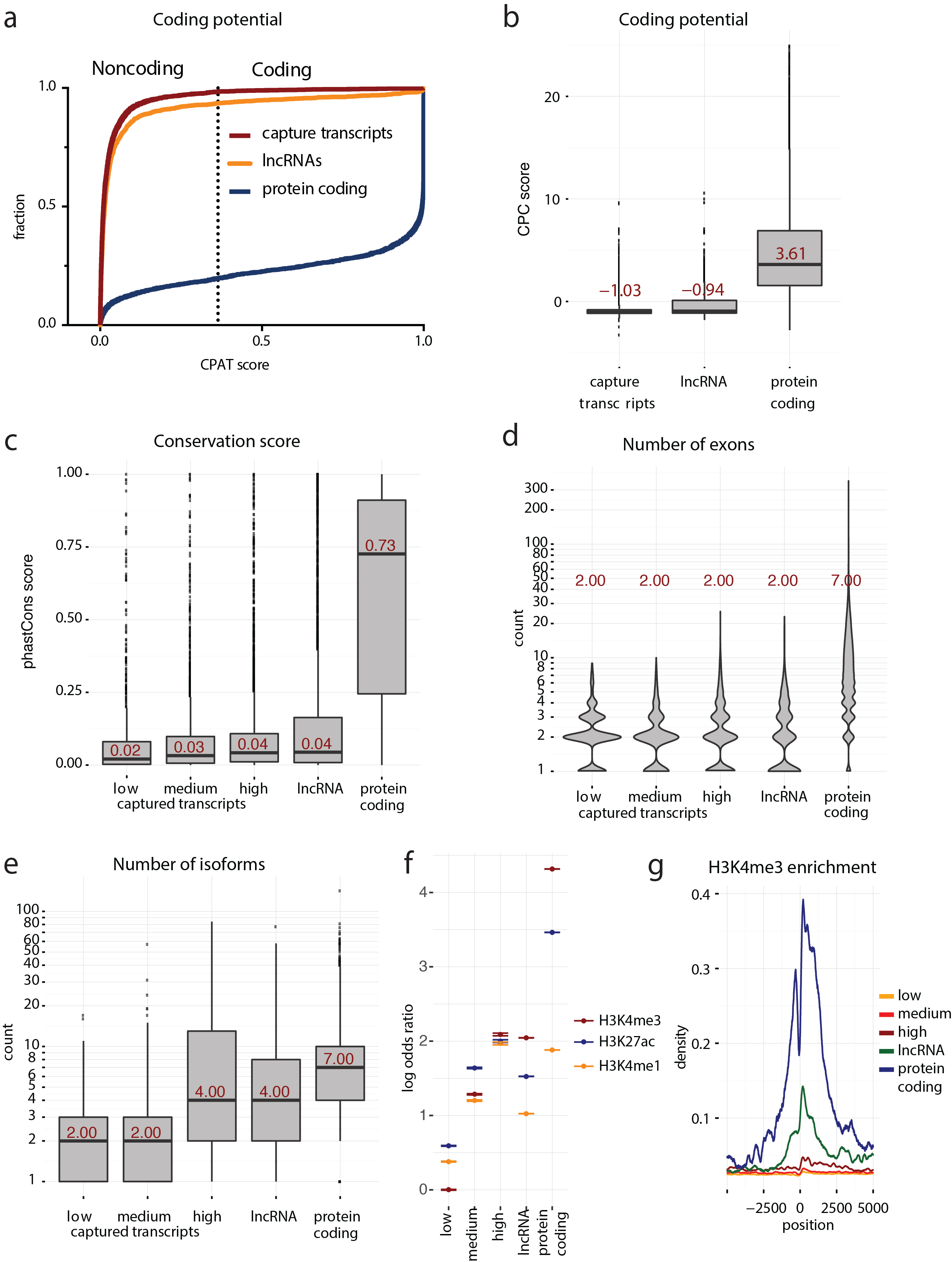


Figure S3. Properties of captured transcripts. A & B. Transcript coding potential as measured by CPAT (A) & CPC (B). Dotted line in A shows CPAT coding potential cutoff. CPC values above 1 are strong evidence for coding potential. C. Conservation score as measured by UCSC phastCons100way. D. Number of exons for captured transcripts compared to GENCODE v.19 genes. E. Number of isoforms for captured transcripts compared to GENCODE v.19. Median per category shown in red. F. Enrichment of epigenetic markers of active transcription around transcription start sites. Log odds ratios and 95% confidence intervals (for details see Enrichment analysis in the Methods section) for enrichment of promoter regions (500bp around start site) withH3K4me3, H3K27ac and H3K4me1 (red, blue and yellow respectively) for captured transcripts expressed in liver (FPKM>1), compared to GENCODE v.19 lncRNAs and protein coding genes. G. Enrichment of H3K4me3 histone mark around the transcription start sites of captured transcripts (expression from 1-10, 10-100 and >100 in yellow, orange, red respectively) compared to GENCODE v.19 lncRNAs and protein coding genes, across all tissues.

Figure S4. Enrichment of CAGE tags around 5’ transcription start sites of captured transcripts and GENCODE genes. Log odds ratios and 95% confidence intervals (for details see Enrichment analysis in the Methods section) for enrichment of promoter regions (500bp around start site) for captured transcripts expressed in liver (FPKM>1), compared to GENCODE v.19 lncRNAs and protein coding genes.


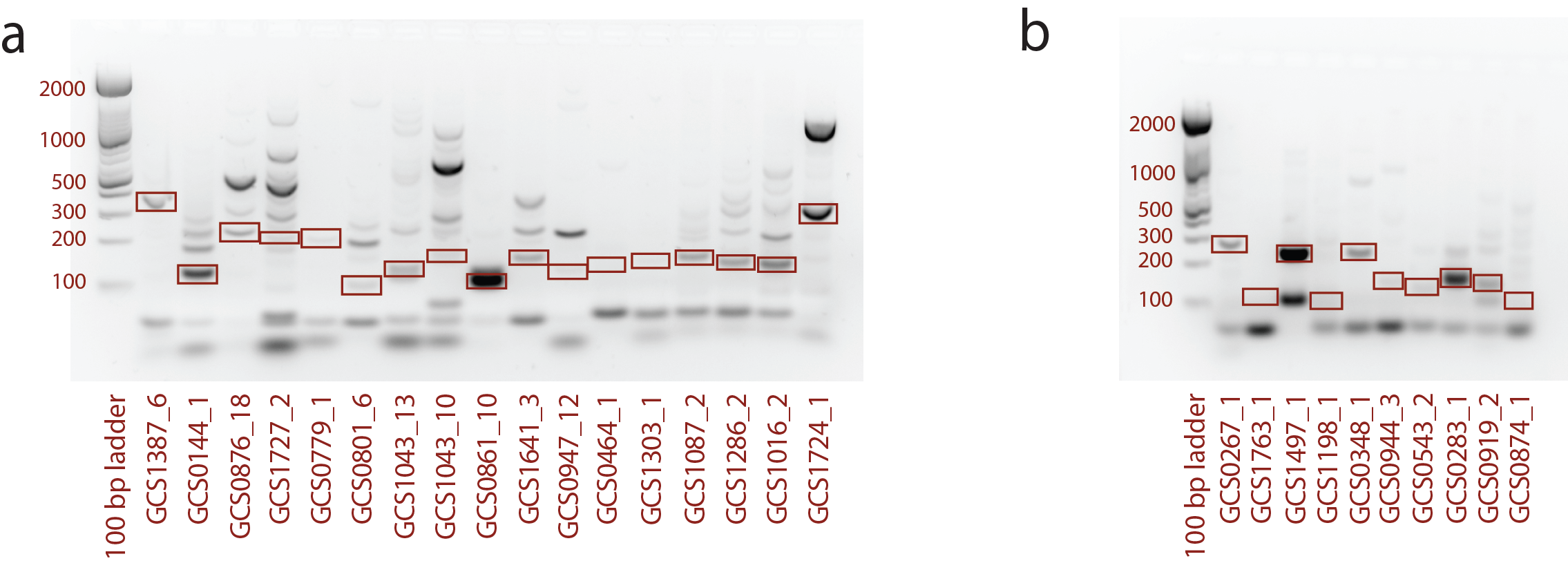


Figure S5. Validation of transcripts with PCR. Out of 30 splice junctions, 27 were successfully validated. Primer design and details in Supplementary table S4.


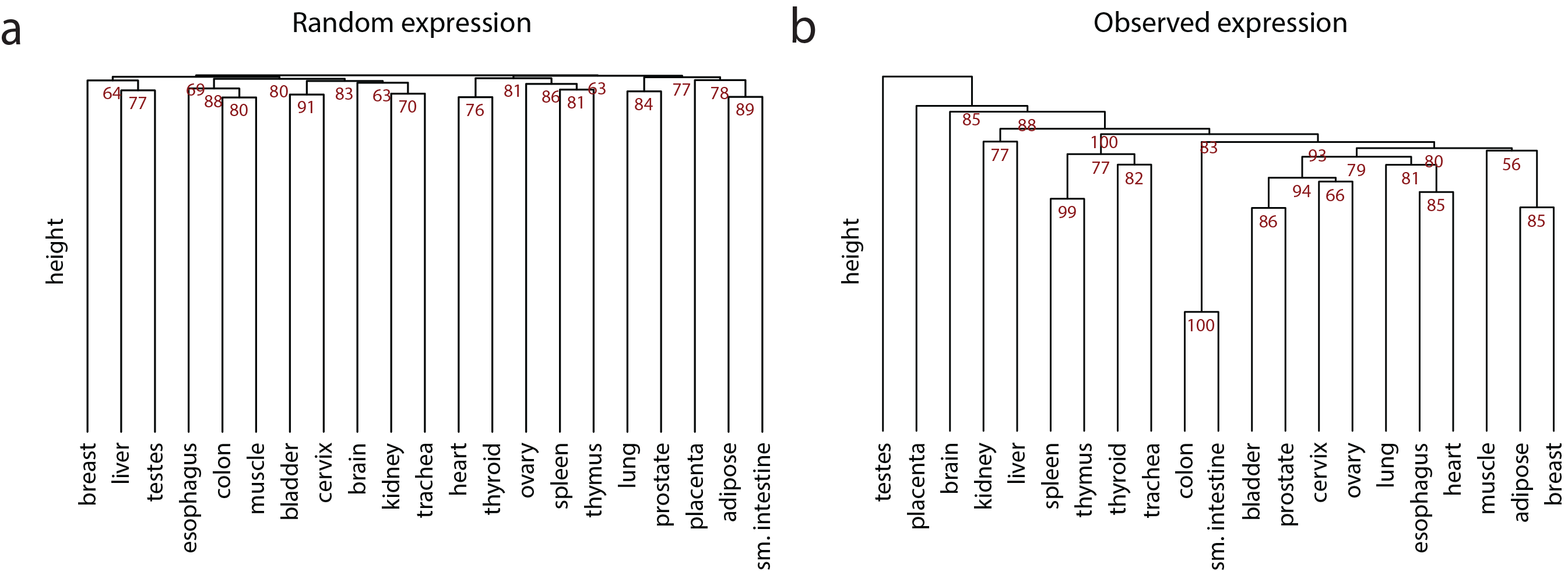


Figure S6. Dendrograms for clustering of tissue specificity of captured transcripts. Significance of individual branches calculated as multiscale bootstrap resampling p-values (given as 1 - p-value, 10.000 bootstraps) with library Pvclust

[84]. Distance is calculated based on correlation, while clustering method is “average”. a. Clustering of randomly permutated expression values. b. Clustering of observed expression values across tissues.

Supplementary Table S5. List of captured transcripts that overlap previously identified lncRNAs that were independently functionally validated.

| **lncRNA** | **Reference** | **lncRNA function** | **Expression** | **Captured transcript** | **Tissue specific expression** | **Haploblock-associated phenotypes** |
| --- | --- | --- | --- | --- | --- | --- |
| CCAT1, PCAT1 | https://www.ncbi.nlm.nih.gov/pubmed/23143645 | promotes the progression of gastric carcinoma | liver | GCS1669 | liver | chronic lymphocytic leukemia;prostate cancer |
| CCDC26 | https://www.ncbi.nlm.nih.gov/pubmed/25928165 | controls myeloid leukemia cell growth | spleen | GCS1684 | spleen | white blood cell count, IBD |
| DLEU1 | https://www.ncbi.nlm.nih.gov/pubmed/23593011 | Cis downregulation of a gene cluster that targets NF-kB | ubiquitous | GCS0496 | ubiquitous | height;pubertal anthropometrics;obesity-related traits;pulmonary function decline |
| LINC00598 | https://www.ncbi.nlm.nih.gov/pubmed/27572135 | modulation of G1 checkpoint through regulation of CCND2 | testis | GCS0535, GCS0490 | adipose | inflammatory bowel disease;ulcerative colitis |
| MAFTRR | https://www.ncbi.nlm.nih.gov/pubmed/25621826 | regulation of T-cell differentiation through regulation of MAF transcription | ubiquitous | GCS0690 | ubiquitous | urate levels;thyroid function |
| PCAT29 | https://www.ncbi.nlm.nih.gov/pubmed/25030374 | androgen-regulated tumor suppressor in prostate cancer | prostate cancer | GCS0611 | prostate | chronic lymphocytic leukemia |
| PTCSC3 | https://www.ncbi.nlm.nih.gov/pubmed/22586128 | tumor supressor for thyroid carcinoma | thyroid | GCS0586 | thyroid | thyroid hormone levels;thyroid cancer |
| SCHLAP1 | https://www.ncbi.nlm.nih.gov/pubmed/24076601 | contributes to the development of prostate cancer by antagonizing the tumor-suppressive functions of the SWI/SNF complex | prostate cancers | GCS0836 | prostate | systemic lupus erythematosus |

Figure S7. Enrichment of ChromHMM marks in captured transcripts compared to other annotated transcript types and randomized transcript locations. ChromHMM marks were downloaded from Roadmap Epigenomics projects and represent the Core 15-state model. Region number and mnemonic are given by the following code: 1 TssA Active TSS, 2 TssFlnk Flanking TSS, 3 TssFlnkU Flanking TSS Upstream, 4 TssFlnkD Flanking TSS Downstream, 5 Tx Strong transcription, 6 TxWk Weak transcription, 7 EnhG1 Genic enhancer1, 8 EnhG2 Genic enhancer2, 9 EnhA1 Active Enhancer 1, 10 EnhA2 Active Enhancer 2, 11 EnhWk Weak Enhancer, 12 ZNF/Rpts ZNF genes & repeats, 13 Het Heterochromatin, 14 TssBiv Bivalent/Poised TSS, 15 EnhBiv Bivalent Enhancer, 16 ReprPC Repressed PolyComb, 17 ReprPCWk Weak Repressed PolyComb, 18 Quies Quiescent/Low.

Figure S8. Transcript models of captured transcripts that overlap independently identified and functionally validated lncRNAs. Red exons denote previously observed splice junctions. Grey boxes mark the captured regions. Histograms on the right of transcript models represent expression of captured transcripts across tissues.


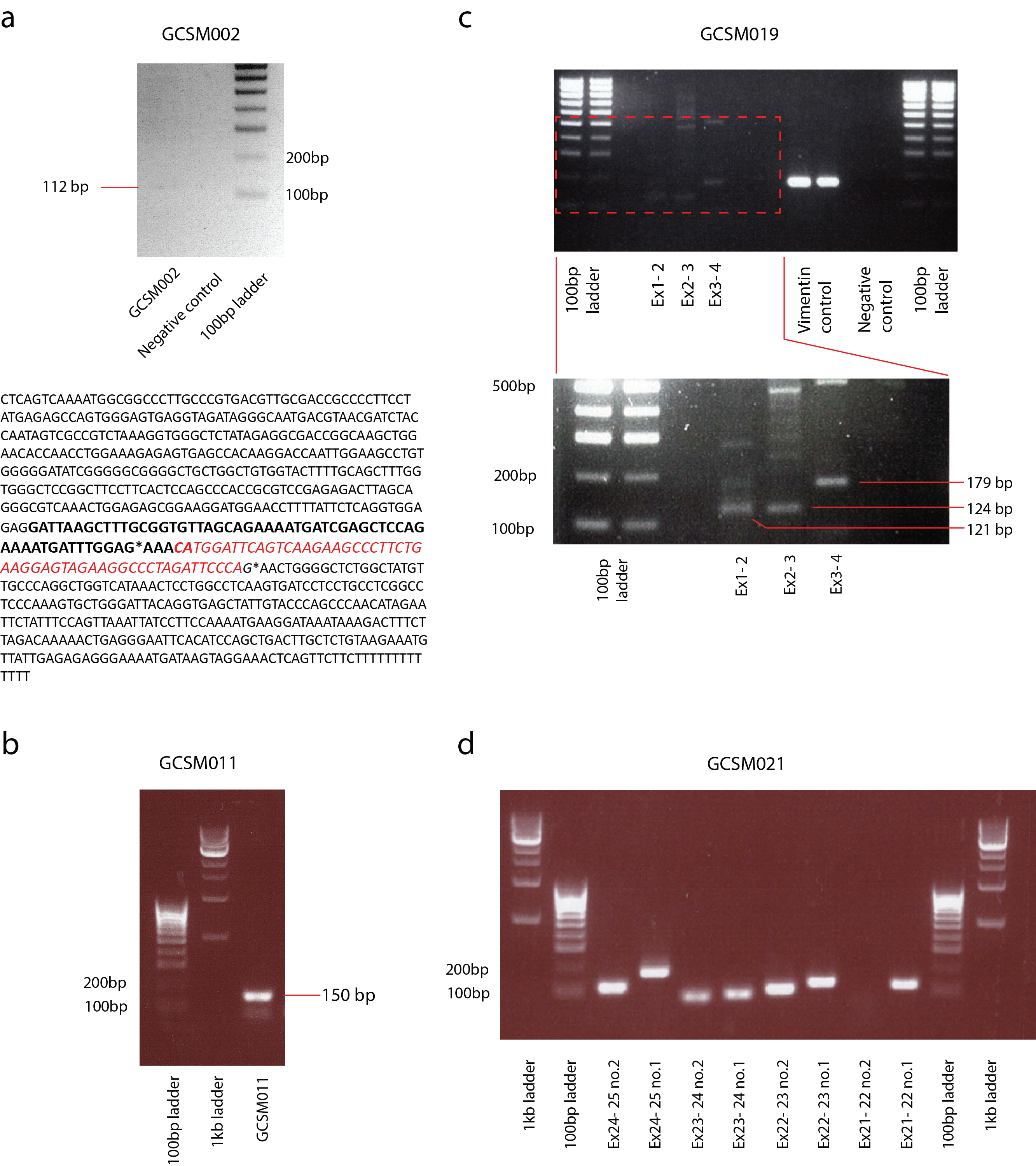


Figure S9. PCR validation of novel Melanoma transcripts. a. GCSM002 bidirectional transcript. Top: PCR for GCSM002, sequenced band of correct size shown. Bottom: Predicted GCSM002 sequence with results of sequencing to confirm exon1-2 splice junction. Position of junctions marked with *. Forward sequence shown in bold, reverse sequence red italics. b. GCSM011 intergenic transcript, sequenced band of correct size shown. c. GCSM019 ACAT1 novel isoform. Top: PCR validations of three novel splice junctions. Vimentin positive control. Bottom: Longer exposure of red boxed region in top panel. Correct sized bands excised for sequencing are indicated. d. GCSM021 NOX4-GRM5 fusion transcript. Validation of fusion transcript by nested PCR, with second round amplicons shown, except Ex21- 22 no.2, which was run as a standalone amplification. All bands were the expected size. Each novel junction was confirmed by sequencing at least one target amplicon. Primer design and details in Sup. table S7.
